# ZPA Regulatory Sequence Variants in Chinese Patients With Preaxial Polydactyly: Genetic and Clinical Characteristics

**DOI:** 10.3389/fped.2022.797978

**Published:** 2022-05-16

**Authors:** Lei Zeng, Jie-Yuan Jin, Fang-Mei Luo, Yue Sheng, Pan-Feng Wu, Rong Xiang

**Affiliations:** ^1^Department of Orthopaedics, Xiangya Hospital, Central South University, Changsha, China; ^2^School of Life Sciences, Central South University, Changsha, China; ^3^Hunan Key Laboratory of Animal Models for Human Diseases, School of Life Sciences, Central South University, Changsha, China; ^4^Hunan Key Laboratory of Medical Genetics, School of Life Sciences, Central South University, Changsha, China

**Keywords:** ZRS, preaxial polydactyly type I, preaxial polydactyly type II, enhancer, SHH

## Abstract

Preaxial polydactyly (PPD) is a common congenital abnormality with an incidence of 0.8–1.4% in Asians, characterized by the presence of extra digit(s) on the preaxial side of the hand or foot. PPD is genetically classified into four subtypes, PPD type I–IV. Variants in six genes/loci [including GLI family zinc finger 3 (*GLI3*), ZPA regulatory sequence (ZRS), and pre-ZRS region] have been identified in PPD cases. Among these loci, ZRS is, perhaps, the most special and well known, but most articles only reported one or a few cases. There is a lack of reports on the ZRS-variant frequency in patients with PPD. In this study, we recruited 167 sporadic or familial cases (including 154 sporadic patients and 13 families) with PPD from Central-South China and identified four ZRS variants in four patients (2.40%, 4/167), including two novel variants (ZRS131A > T/chr7:g.156584439A > T and ZRS474C > G/chr7:g.156584096C > G) and two known variants (ZRS428T > A/chr7:g.156584142T > A and ZRS619C > T/chr7:g.156583951C > T). ZRS131A > T and ZRS428T > A were detected in PPD I cases and ZRS474C > G and ZRS619C > T combinedly acted to cause PPD II. The detectable rate of ZRS variants in PPD I was 1.60% (2/125), while PPD II was significantly higher (9.52%, 2/21). Three bilateral PPD cases harbored ZRS variants (13.64%, 3/22), suggesting that bilateral PPD was more possibly caused by genetic etiologies. This study identified two novel ZRS variants, further confirmed the association between ZRS and PPD I and reported a rare PPD II case resulted from the compound heterozygote of ZRS. This investigation preliminarily evaluated a ZRS variants rate in patients with PPD and described the general picture of PPD in Central-South China.

## Introduction

Preaxial polydactyly (PPD) is a common congenital abnormality with an incidence of 0.8–1.4% in Asians, characterized by the presence of extra digit(s) on the preaxial side of the hand or foot (1). Severity varies from mere broadening of the distal phalanx with slight bifurcation at the tip to full duplication of the thumb, including the metacarpals (2). PPD is genetically classified into four subtypes, PPD type I–IV (Table 1) (3). PPD I (OMIM 174400) indicates the duplication of one or more of the skeletal components of biphalangeal thumbs, which is the most common subtype in many populations (2). PPD II (OMIM 174500) refers to isolated triphalangeal thumbs or the thumb duplication with triphalangeal components (4). PPD III (174600) is also known as index finger polydactyly. Thumbs of PPD III cases are replaced by one or two index fingers (5). PPD IV (174700) is polysyndactyly of the thumb (6).

**TABLE 1:** Classification of PPD and their clinical features and causative genes/loci.

Subtype	OMIM	Clinical features	Heredity	Gene/Locus
PPD I	174400	The duplication of one or more of the	AR	*GLI1*
		skeletal components of biphalangeal thumbs	AR	Serine/threonine kinase like domain containing 1 (*STKLD1*)
			AD	ZRS
PPD II	174500	Isolated triphalangeal thumb or thumb duplication	AD	ZRS
		with a triphalangeal component	AD	pre-ZRS
PPD III	174600	Thumbs replaced by one or two index fingers	–	–
PPD IV	174700	Polysyndactyly of the thumb	AD	GLI family zinc finger 3 (GLI3)

*PPD, preaxial polydactyly; AD, autosomal dominant; AR, autosomal recessive.*

Currently, only six genes/loci [*GLI1*, *GLI3*, serine/threonine kinase like domain containing 1 (*STKLD1*), ZPA regulatory sequence (ZRS), pre-ZRS region, and a deletion of 240 kb from the sonic hedgehog signaling molecule (*SHH*) promoter] have been identified in isolated PPD cases and ZRS is, perhaps, the most special and well known (7–12). ZRS, the zone of polarizing activity (ZPA) (located in the posterior region of the limb bud) regulatory sequence, is a limb-specific enhancer of *SHH*, which is located nearly 1 Mb from *SHH* and within intron 5 of *Limb development membrane protein 1 (LMBR1)* (4). ZRS can promote the expression of SHH in ZPA during the limb development. SHH diffuses from ZPA (posterior mesoderm) to anterior region of limb bud and there is no SHH in anterior region. The graded distribution of SHH determines the finger pattern. ZRS variants would alter the expression of SHH and cause limb deformities. ZRS variants and duplications had been shown to cause PPD I, PPD II, Werner mesomelic syndrome (WMS) (OMIM 188770), and other limb deformities (such as mirror-image polydactyly and radial ray deficiency) (12–16). The correlation between PPD and ZRS is definite, but most articles only reported one or a few cases, especially in PPD II cases. There is a lack of reports on the ZRS-variant frequency in patients with PPD.

In this study, we recruited 167 sporadic or familial cases with PPD from Central-South China. We identified four ZRS variants in four PPD cases (4/167, 2.40%), including two novel variants (ZRS131A > T/chr7:g.156584439A > T and ZRS474C > G/chr7:g.156584096C > G) and two known variants (ZRS428T > A/chr7:g.156584142T > A and ZRS619C > T/chr7:g.156583951C > T). This study preliminarily investigated the ZRS variant rate in patients with PPD living in Central-South China, expanded the spectrum of ZRS variants, furthered our understanding of PPD, and contributed to genetic diagnosis and counseling of patients with PPD.

## Materials and Methods

### Patients and Subjects

This study was approved by the Review Board of Xiangya Hospital of Central South University. A total of 167 sporadic or familial PPD cases admitted to the Department of Orthopaedics of Xiangya Hospital were recruited. They were all from Central-South China, especially Hunan province. Almost subjects were preschoolers and informed consent forms were obtained from the patients and their guardians. All the subjects and their guardians consented to participate in this study and to publication of the images. Blood was collected from patients and their blood relations.

### Deoxyribonucleic Acid Extraction

Peripheral blood samples were collected from patients and their family members to extract genomic DNA by the DNeasy Blood and Tissue Kit (Qiagen, Valencia, CA, United States).

### Variant Screening

The highly conserved 774-bp region of the ZRS (chr7: 156583796-156584569, hg19) was obtained from the National Center for Biotechnology Information (NCBI) database^1^ and primers were designed by Integrated DNA Technologies (IDT)^2^ (Table 2) (17). PCR was operated to amplify the target sequences by CFX384 Touch PCR Amplifier (Bio-Rad, Hercules, CA, United States). PCR product sequences were determined using the ABI 3100 Genetic Analyzer (Thermo Fisher Scientific, Waltham, MA, United States) by Sanger sequencing performed by Boshang Biotechnology Co., Ltd. (Shanghai, China). For patients who were identified ZRS variants, further genetic screening (using PCR and Sanger sequencing) was used to detect whether they harbored pathogenic variants in *GLI3* (NM_000168.6, NP_000159.3), *GLI1* (NM_005269.3, NP_005260.1), *STKLD1* (NM_153710.5, NP_714921.4), or pre-ZRS. Their primer pairs were also designed by IDT (Table 2).

**TABLE 2:** Primer pairs of ZPA regulatory sequence (ZRS), *GLI3*, *GLI1*, *STKLD1*, and pre-ZRS.

Primer	Sequences (5′→3′)	Primer	Sequences (5′→3′)
ZRS 1f	GGAGGTATAACCTCTGGCCAGTG	ZRS 1r	CGCTTCCACCTGGTCAGTCC
ZRS 2f	CCAGAGCGTAGCACACGGTC	ZRS 2r	CAATTTATGGATCATCAGTGGC
ZRS 3f	TCAGGCCTCCATCTTAAAGAG	ZRS 3r	GAAATGGTTATGGATCAGAAAGT

GLI3 1f	GAAAGTTGATGGCTCTGTTGTTT	GLI3 1r	CAGGTGCAAACGCTCAATTC
GLI3 2f	GCTCTCAAAGTTGCTGTGAATG	GLI3 2r	TGGGAAAGAAGTAGGGAAAGTAAG
GLI3 3f	CAGTACCTCACAGAGCTTCATAAC	GLI3 3r	CAGTGAACCCACGAACAGATAG
GLI3 4f	TTCTCATGGAAGAAGCCATAGG	GLI3 4r	CTTTATACACGTCCCGAGTGAG
GLI3 5f	TCTGAGATGCCTCAAGAGAAAC	GLI3 5r	GGGTCTCAGGATGTCCAAAT
GLI3 6f	GCAAGTTGCCAGCTTCTTATC	GLI3 6r	TTTGACCTGCCTCTTGGTATAG
GLI3 7f	TTAGGTCTGCGTGTATGTGTG	GLI3 7r	GACATGGGATGCAGGTTACA
GLI3 8f	TGGTACTGCTCCTTGTTGATG	GLI3 8r	ACTGCCTGTGTTTGCTTCT
GLI3 9f	CCTCCTGTTGTGTCTGATTCTT	GLI3 9r	GTCATAAAGCCCTCTCCAGTTC
GLI3 10f	AGGAAGCATGCATACACAGTTA	GLI3 10r	CATCAGTTTGCACAGCTCTTATG
GLI3 11f	AACTTGGAGGGCGTGTTAG	GLI3 11r	CGGGATAGTTCTTTGTTTCCTTATG
GLI3 12f	TACCTCGTTCTTGTGGGATTTG	GLI3 12r	CTTCTCTGCCTTGACGGTTT
GLI3 13f	ATTGGCTCCCTTTCCTTGAC	GLI3 13r	CAGATGCATGGTCTGATGTAGAA
GLI3 14-1f	TGGTCTCTCCCTTTCTCTGT	GLI3 14-1r	TCAGGCTCATCCTCTCCAT
GLI3 14-2f	CAGCAGTACCGCCTCAAG	GLI3 14-2r	TCGTTCAGGTTGGCATCAG
GLI3 14-3f	CAGTCCCGAAACTTCCACTC	GLI3 14-3r	GCCTTACAGGGCTGTTCAT
GLI3 14-4f	CCCATTCAGTGGAACGAAGT	GLI3 14-4r	GCCCTTGGTAGATGTTGATGT
GLI3 14-5f	AGATGCTTGGGCAGATTAGTG	GLI3 14-5r	GCTGGCGTCTGAAATAGAGAA
GLI3 14-6f	CCATCCGCTGTGCTCTAATC	GLI3 14-6r	TCCGTTGGTTGCAGTCTTT

GLI1 1f	ATTCCGTGGCAGATGTCTTAG	GLI1 1f	CTGGAATGGGAATGGAGGATAC
GLI1 2f	CCCATGCCAGTTTCCTATCTAC	GLI1 2f	CCTCACATCTCCAAGCATCTC
GLI1 3f	CATAGGTTAGGTGCATGGAGAG	GLI1 3r	CTCAGGAAGGATTGGGCTATTT
GLI1 4f	TCAAGCCCTCAAACTACCTAAC	GLI1 4r	CTCAGACTACACTGGTGAATGG
GLI1 5 + 6f	CATCCCATTCACCAGTGTAGTC	GLI1 5 + 6r	GGAAGAGGCAGGAGCAATATC
GLI1 7f	GGAAGACCTGAGATGTGAGATATG	GLI1 7r	GAGAGCCCTGATTTAGGAAGAG
GLI1 8f	TGTGTGTCCTGTTGGAGATTG	GLI1 8r	GTAGGAGGAGGAGTGGTTAAGT
GLI1 9-1f	CTCCATCCTCCTTACTTCCTTTG	GLI1 9-1r	CTTGGGCTCCACTGTAGAAAT
GLI1 9-2f	CCACTCTCCACTCAACAGAAG	GLI1 9-2r	GAATGGATGGGTTGGGAAGTA
GLI1 10f	TGCTTAGCCCTTTCTACACTTAC	GLI1 10r	TGACTTCCTCCTCTCAACCT
GLI1 11-1f	AGGAGGCAGGGTGAAATTTAG	GLI1 11-1r	AGAGTATCAGTAGGTGGGAAGT
GLI1 11-2f	TACCTCCCAACCTCTGTCTAC	GLI1 11-2r	GCCCTATGTGAAGCCCTATTT
GLI1 11-3f	CTACCAGAGTCCCAAGTTTCTG	GLI1 11-3r	GCGATCTGTGATGGATGAGATT

STKLD1 1f	TACGCGGTCGCTACTGAT	STKLD1 1r	CCCACGCCCTCAAATACTC
STKLD1 2f	AGGGATACAGGGTTGTAGAAGA	STKLD1 2r	GATTAGTCTCCGCAAGTGTCAG
STKLD1 3f	GTTGGTTGTGGTTGTGGTAATG	STKLD1 3r	AACTGGTGCTGATGCTCTATC
STKLD1 4f	GTTGGGATGTGTGACAGAGAAG	STKLD1 4r	CCTATGAGACTATGCACCGAAAG
STKLD1 5f	AGAGAGAGGAAGCTGAAGGT	STKLD1 5r	CCTCGAGGCACACATTTAAGA
STKLD1 6f	CAAGATGCAAGGAGAGGATACA	STKLD1 6r	GCTTGAGACCACTTGGAAGA
STKLD1 7f	TTTGTGGAGGAGAGGAGGAT	STKLD1 7r	AGGAGGTCTCTTTGGAGTTTAC
STKLD1 8f	TGGCTCCAGATCAACACAAA	STKLD1 8r	CACTGCTGTCATTATCCTGCTA
STKLD1 9f	GGTCTCTGGGCATTCTTGTAG	STKLD1 9r	GTGCTTGTATTAGGGTGGAGAG
STKLD1 10f	GAGAGACCCTGCCAAATGAA	STKLD1 10r	GTTGGGAGCTATGGAGGATATTT
STKLD1 11f	CATCATCTGTGTGCTCCAAGAC	STKLD1 11r	GCCTCCACGCTGCAATAAA
STKLD1 12f	GACCTAGCGCTAATCCTCATTG	STKLD1 12r	CCTAGAAGATGGCCTAGAAGGT
STKLD1 13f	CATTAGGCCACAGGGATTCA	STKLD1 13r	AGGATGCGACCAGCATTT
STKLD1 14f	GTAGTGGGATGGCAGCTATTG	STKLD1 14r	TGGGCAAGAAGTCCTGAAAC
STKLD1 15-16f	GTTGTCGTTAGCTGGAGGAA	STKLD1 15-16r	ACCTGGCAGATGTAACTGATG
STKLD1 17f	TTCTTGCATGGTCCTGTTCA	STKLD1 17r	GCCAAATGAGTGGGAAGTTTAAG
STKLD1 18f	CCCACTTAAACTTCCCACTCAT	STKLD1 18r	CAGGAAACTCTTTGGAGAGGTC

pre-ZRS 1f	GGAAGTGCTGCTTAGTGTTAGT	pre-ZRS 1r	GTTCCCATACGCCCAGATTT
pre-ZRS 2f	GCTGTGATACTTCAGCTTCCT	pre-ZRS 2r	GCCATAATTTAGTGCCCTCCTA
pre-ZRS 3f	AAATCTGGGCGTATGGGAAC	pre-ZRS 3r	CCTGGTAGACAGGTACTGTTAGA
pre-ZRS 4f	TGGATCTAGGAGGGCACTAAA	pre-ZRS 4r	CAGAGGCCTGAACTATCAAACA
pre-ZRS 5f	ACATCAGGAGAACTTGTGTAGG	pre-ZRS 5r	CCAACCAAGGGTGAGTAGTT
pre-ZRS 6f	ACTGGCTGTAATACTACTCCAATAC	pre-ZRS 6r	AACAATCTTACTGCCTTTGATGTG
			

### Prediction of Pathogenicity

MutationTaster^3^ was applied for predicting the pathogenicity of variants. GnomAD^4^ was used to annotate the minimum allele frequency (MAF) of variants. ZRS sequences of species from Evgeny et al. (18) were used to compare the conservation of variant sites (18).

## Results

We recruited 167 cases with PPD from Central-South China, including 154 sporadic patients and 13 families, named as PPD001–PPD167 depending on the order of recruitment. Among these cases, almost subjects (154/167, 92.22%) were Han Chinese and 148 patients had isolated PPD (Table 3). Based on PPD subtypes to divide subjects, 125 patients (74.85%) revealed PPD I, 21 patients (12.57%) had PPD II, and only four cases exhibited PPD III (1/167, 0.60%) or PPD IV (3/167, 1.80%). The rest of PPD subjects (17 cases, 10.18%) presented other organ malformations, such as congenital heart disease, radial ray deficiency, and anal atresia. There were 103 male patients (61.68%) and 64 female patients (38.32%). Most subjects were younger than 3 years old. Except 19 cases without clinical details, the overwhelming majority of PPD I/II was unilateral (109/127, 85.83%), in which PPD, on the right hand, accounted for almost two-thirds (72/109, 66.06%; Table 3).

**TABLE 3:** Characteristics and clinical phenotypes of all the subjects.

	PPD I	PPD II	PPD III	PPP IV	Others*	Total	PPD with ZRS variants
Age (years)	3.326 ± 0.518	2.730 ± 0.695	0.9	3.400 ± 0.513	6.029 ± 1.904	3.529 ± 0.446	1.750 ± 0.777
Gender	78 M; 47 F	12 M; 9 F	1 M; 0 F	2 M; 1 F	10 M; 7 F	103 M; 64 F	3 M (2.91%); 1 F (1.56%)
Ethnicity (Han)	114	19	1	3	17	154	4
Other ethnicities**	11	2	0	0	0	13	0
Number	125	21	1	3	17	167	4
Proportion	74.85%	12.57%	0.60%	1.80%	10.18%	100.00%	2.40%
Unilateral	32 L; 63 R	5 L; 9 R	0	0	–	37 L; 72 R	0 L (0.00%); 1 R (1.39%)
Bilateral	12	6	1	3	–	22	3 (13.64%)
Cases without details	18	1	0	0	–	19	0
Familial/sporadic	8/117	3/18	0/1	1/2	1/16	13/154	1/3
Isolated/syndromic	125/0	21/0	1/0	1/2	0/17	148/19	4/0
ZRS variants detection rate	1.60%	9.52%	0.00%	0.00%	0.00%	2.40%	–

PPD, preaxial polydactyly; M, male; F, female; L, left thumb involved; R, right thumb involved. *PPD with multiple organ malformations, such as congenital heart disease, radial ray deficiency, anal atresia. **Other ethnicities include Tujia nationality, Miao nationality, and Hui nationality.

**FIGURE 1:**
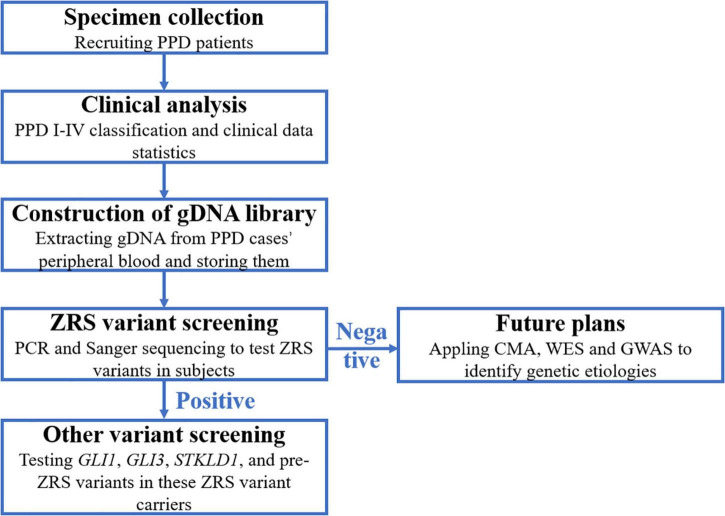
The flow diagram of this study. PPD, preaxial polydactyly; CMA, chromosomal microarray analysis; WES, whole-exon sequencing; GWAS, genome-wide association study.

In accordance with the flow diagram (Figure 1), we identified four ZRS variants in four patients (PPD003, PPD029, PPD116, and PPD154; Table 4). The detectable rate of ZRS variants in PPD I was 1.60% (2/125), while PPD II was significantly higher (2/21, 9.52%). Three of these four patients were with bilateral thumbs involvement, occupying 13.64% of bilateral PPD (3/22). None ZRS variant was identified in patients with left PPD, although they were more than one-third total subjects (37.72%, 63/167).

**TABLE 4:** Phenotypes and genotypes of patients with PPD with ZRS variants.

Patient	Age (years)	Gender	Phenotype	ZRS variant	Location (hg19)	MutationTaster	GnomAD
PDD003	1	M	Bilateral PPD with triphalangeal thumb on the right hand	ZRS428T > A	Chr7:156584142	D	0.00006
PDD116	0.5	M	Bilateral PPD I				
PDD029	4	F	Bilateral triphalangeal thumbs	ZRS474C > G	Chr7:156584096	D	–
				ZRS619C > T	Chr7:156583951	D	0.00000
PDD154	1.5	M	PPD I on the right hand	ZRS131A > T	Chr7:156584439	D	–

*PPD, preaxial polydactyly; M, male; F, female; D, disease causing.*

### PPD029 Family

**FIGURE 2:**
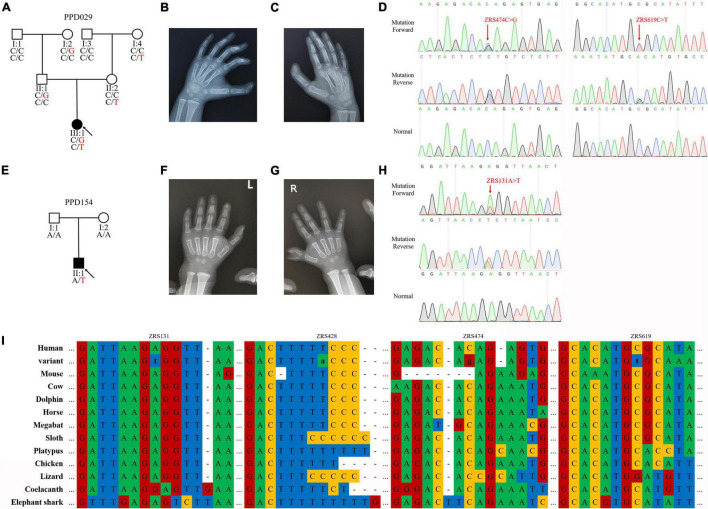
ZPA regulatory sequence (ZRS) variants identified in preaxial polydactyly (PPD) families. **(A,E)** Pedigrees of PPD families (PPD029 and PPD154). “PPD029” and “PPD154” were PPD family numbers. Squares indicate male family members and circles indicate female family members. The black symbols represent the affected members and arrows indicate probands. Genotypes are identified by letters and a slash, with red representing variants. **(B,C,F,G)** Symptoms of patients. PPD029 had bilateral triphalangeal thumbs and PPD154 exhibited PPD I on the right hand. **(D,H)** Sequencing results of ZRS variants using Sanger sequencing. **(I)** Species conservation analysis of mutant base sites in ZRS.

The proband of PPD029 (III:1) was a 4-year-old girl, who presented bilateral triphalangeal thumbs (Figures 2A–C). She harbored compound heterozygous variants in ZRS (ZRS474C > G/chr7:g.156584096C > G and ZRS619C > T/chr7:g.156583951C > T) without *GLI3*, *GLI1*, *STKLD1*, or pre-ZRS variants (Figure 2D). ZRS474C > G was inherited from her father (II:2) and another variant was from her mother (II:3). Other family members without variants or with only one variant were unaffected.

### PPD154 Family

The proband of PPD154 (II:1) was a boy with PPD I on the right hand (Figures 2E–G). He was admitted to our hospital for operative treatment at his age of 1.5 years. We identified a *de novo* variant in ZRS (ZRS131A > T/chr7:g.156584439A > T) in this patient and did not find suspicious variants in *GLI3*, *GLI1*, *STKLD1*, and pre-ZRS (Figure 2H). His parents were unaffected.

### PPD003 Family and PPD116 Family

Known ZRS variant (ZRS428T > A/chr7:g.156584142T > A) was identified in two families with PPD II (PDD003) or PPD I (PDD116). Four variants identified in this study were highly evolutionarily conserved and were predicted to be disease-causing by MutationTaster (Figure 2I and Table 4).

## Discussion

Polydactyly is the most common limb malformation in China and PPD is over half (data from National Stocktaking Report on Birth Defect Prevention)^5^. PPD I is the most common subtype and PPD III is rarest (2, 19). In this study, 125 cases (74.85%, 125/167) had PPD I and only one patient (1.80%, 1/167) was diagnosed with PPD III. 17 patients with PPD (10.18%, 17/167) had other organ malformations, including congenital heart disease, radial ray deficiency, and anal atresia. These complications were relatively frequent in patients with PPD. Male patients with PPD are approximately twice as many as female (19). In this study, the proportion of male patients was 61.68% (103/167). This study showed that overwhelming majority of PPD I/II were unilateral (85.83%, 109/127), in which PPD, on the right hand, accounted for almost two-thirds (66.06%, 72/109), consistent with previous studies (19, 20).

In this study, we tested ZRS variants in 167 patients with PPD and identified unique variants (MAF ≤ 0.05) in four cases (2.40%, 4/167). The detectable rate of ZRS variants in PPD I was 1.60% (2/125), while PPD II was significantly higher (9.52%, 2/21). Indeed, most known ZRS variants are identified in PPD II cases [data from the human gene mutation database (HGMD)]^6^. In this study, three ZRS variants were associated with bilateral PPD and 13.64% bilateral PPD cases (3/22) harbored ZRS variants, suggesting that bilateral PPD was more possibly caused by genetic etiologies. Compared with that no ZRS variant was detected by Xiang et al. (20) in 82 Chinese patients with PPD I/II or Rao et al. (21) in 72 Chinese patients with PPD, our identification was fortunate (20, 21). For the remaining 163 cases, we planned to applied chromosomal microarray analysis (CMA), whole-exon sequencing (WES), and genome-wide association study (GWAS) to detect their genetic etiologies. Furthermore, environmental factors, such as alcohol, are causes of limb deformities (22).

Of these four variants, ZRS131A > T and ZRS474C > G were novel and ZRS428T > A and ZRS619C > T had been reported in patients with PPD II (4, 15). ZRS428T > A was identified in both the patients with PPD I (PPD116) and PPD II (PPD003), suggesting the variability of ZRS428T > A-related clinical phenotypes. ZRS131A > T was identified in a sporadic case with PPD I (PPD154). Generally, ZRS variants are associated with PPD II and ZRS was first linked with PPD I by Xu et al. (12). Our report may be the second case worldwide, further demonstrating the correlation between ZRS and PPD I.

PPD029 was a rare case. We found that the proband harbored the compound heterozygote of ZRS (ZRS474C > G and ZRS619C > T). Given that Jacob et al. (23) reported ZRS variant carriers with minor anomalies and underlined the importance of accurate clinical examination in mild triphalangeal thumb families, we carefully checked the phenotypes of her family members with one ZRS variant and did not find any limb defects (cannot completely exclude the possibility of an extremely subtle anomaly) (23). We reasoned that PPD in this family was attributed to combinedly acting by these two variants. PPD II is an autosomal dominant disease and our description indicated that PPD II individuals can be affected with a pattern of autosomal recessive inheritance. A previous study indicated that compared with a heterozygous variant in ZRS (ZRS402C > T), the homozygote led to more severe phenotypes, WMS, manifesting the superimposed effect of ZRS variants and our detection demonstrated again this phenomenon (24). ZRS619C > T had been reported by Mohammad et al. (15) in a Saudi Arabian family presented with variable preaxial deformities of the upper limbs including isolated triphalangeal thumb, PPD, preaxial syndactyly, and absent thumb and radius (15). Some family members suffered from renal agenesis and congenital heart disease. The variant (ZRS619C > T) showed obvious phenotypic heterogeneity in the Saudi Arabian family, whereas the variant was unable to alone trigger PPD in PPD029 family. It suggested that the pathogenicity of ZRS variants may be affected by ethnic difference, individual variation, and/or environmental factor.

ZPA regulatory sequence is a limb-specific enhancer of *SHH*, which induces the expression of SHH within ZPA (25). SHH expends from posterior mesoderm to anterior region of limb buds and lacks within the anterior-proximal. The expression gradient of SHH is crucial in establishing the number and the identity of the digits during anteroposterior patterning of the limb (26). Duplications involved ZRS or gain-of-function variants in ZRS would promote the expression of SHH in ZPA and then trigger the ectopic expression within the anterior region, where proliferation of mesenchymal cells is increased to cause PPD I/II (27). For four ZRS variants identified by us, their biological functions were not clarified and further studies needed to be performed. But, we predicted the pathogenicity of these four ZRS variants and analyzed their conservation. GnomAD showed that these variants were absent from controls or extremely rare. Thus, we highly suspected that these ZRS variants were their genetic etiologies, which should be further investigated.

## Conclusion

In summary, we recruited 167 sporadic or familial cases with PPD from Central-South China and identified four ZRS variants (ZRS131A > T/chr7:g.156584439A > T, ZRS428T > A/chr7:g.156584142T > A, ZRS474C > G/chr7:g.156584096C > G, and ZRS619C > T/chr7:g.156583951C > T) in four patients with PPD (2.39%). Our description about epidemiological investigation of PPD helped us to understand the general picture of PPD in Central-South China. Our detection of two novel ZRS variants not only enrich the genetic map of PPD, but also contributed to genetic diagnosis and counseling of patients with PPD. Furthermore, we reported two patients with PPD I harboring ZRS variants further supporting the link between ZRS and PPD I and a PPD II case caused by the compound heterozygote in ZRS contributing to our understanding of PPD II and its genetic mechanism.

## Data Availability Statement

The original contributions presented in the study are included in the article/supplementary material, further inquiries can be directed to the corresponding authors.

## Ethics Statement

The studies involving human participants were reviewed and approved by the Review Board of Xiangya Hospital of Central South University. Written informed consent to participate in this study was provided by the participants’ legal guardian/next of kin. Written informed consent was obtained from the individual(s), and minor(s)’ legal guardian/next of kin, for the publication of any potentially identifiable images or data included in this article.

## Author Contributions

LZ performed the acquisition, analysis, and interpretation of the data. J-YJ contributed to conception and design, carried out the analysis, and interpretation of the data. F-ML and YS carried out the analysis and interpretation of the data. P-FW contributed to conception and design and wrote the original draft. RX revised the draft and finally approved the final version of the manuscript. All authors contributed to the article and approved the submitted version.

## Conflict of Interest

The authors declare that the research was conducted in the absence of any commercial or financial relationships that could be construed as a potential conflict of interest.

## Publisher’s Note

All claims expressed in this article are solely those of the authors and do not necessarily represent those of their affiliated organizations, or those of the publisher, the editors and the reviewers. Any product that may be evaluated in this article, or claim that may be made by its manufacturer, is not guaranteed or endorsed by the publisher.

## Footnotes

1
https://www.ncbi.nlm.nih.gov/gene/105804841

2
http://sg.idtdna.com/Primerquest/Home/Index

3
http://www.mutationtaster.org/

4
http://gnomad-sg.org/

5
https://wenku.so.com/d/8c145f3c9e9b4892c02bf7e70aa83d01

6
http://www.hgmd.cf.ac.uk/ac/search.php
